# Pannexin 1 Channels Play Essential Roles in Urothelial Mechanotransduction and Intercellular Signaling

**DOI:** 10.1371/journal.pone.0106269

**Published:** 2014-08-29

**Authors:** Hiromitsu Negoro, Marcia Urban-Maldonado, Louis S. Liou, David C. Spray, Mia M. Thi, Sylvia O. Suadicani

**Affiliations:** 1 Department of Urology, Albert Einstein College of Medicine, Bronx, New York, United States of America; 2 Dominick P. Purpura Department of Neuroscience, Albert Einstein College of Medicine, Bronx, New York, United States of America; 3 Department of Urology, Cambridge Health Alliance, Cambridge, Massachusetts, United States of America; 4 Department of Orthopaedic Surgery, Albert Einstein College of Medicine, Bronx, New York, United States of America; Institut Curie, France

## Abstract

Urothelial cells respond to bladder distension with ATP release, and ATP signaling within the bladder and from the bladder to the CNS is essential for proper bladder function. In other cell types, pannexin 1 (Panx1) channels provide a pathway for mechanically-induced ATP efflux and for ATP-induced ATP release through interaction with P2X_7_ receptors (P2X_7_Rs). We report that Panx1 and P2X_7_R are functionally expressed in the bladder mucosa and in immortalized human urothelial cells (TRT-HU1), and participate in urothelial ATP release and signaling. ATP release from isolated rat bladders induced by distention was reduced by the Panx1 channel blocker mefloquine (MFQ) and was blunted in mice lacking Panx1 or P2X_7_R expression. Hypoosmotic shock induced YoPro dye uptake was inhibited by MFQ and the P2X_7_R blocker A438079 in TRT-HU1 cells, and was also blunted in primary urothelial cells derived from mice lacking Panx1 or P2X_7_R expression. Rinsing-induced mechanical stimulation of TRT-HU1 cells triggered ATP release, which was reduced by MFQ and potentiated in low divalent cation solution (LDPBS), a condition known to enhance P2X_7_R activation. ATP signaling evaluated as intercellular Ca^2+^ wave radius was significantly larger in LDPBS, reduced by MFQ and by apyrase (ATP scavenger). These findings indicate that Panx1 participates in urothelial mechanotransduction and signaling by providing a direct pathway for mechanically-induced ATP release and by functionally interacting with P2X_7_Rs.

## Introduction

ATP plays important roles in sensory and motor functions of the urinary bladder. ATP co-released with acetylcholine from parasympathetic fibers can directly excite the bladder detrusor muscle, and ATP released from the urothelium in response to stretch of the bladder wall as it fills with urine has been proposed to convey information to the CNS regarding the degree of bladder distension by activating suburothelial afferent nerve fibers [1], [2].

The participation of urothelial-derived ATP and purinergic receptors (P2Rs) in the bladder mechanosensory and transduction systems is supported by an ever growing body of evidence, starting with the identification of a population of suburothelial afferents that express purinergic P2X_3_ receptors [3]–[5], observations that desensitization of P2X receptors or administration of P2R blockers significantly depress the activity of the bladder afferents in response to distension [4], [6], and demonstrations that stretch-induced urothelial ATP release is not altered in P2X_3_R-null mice but absence of this receptor results in marked bladder hyporeflexia with the animals displaying increased voiding volume and reduced voiding frequency [7].

Urothelial ATP release has been shown to be increased in humans with several bladder conditions, such as interstitial cystitis [8], irritative voiding from benign prostatic hyperplasia [9], painful bladder syndrome [10], bladder overactivity [11] and also in animal models of spinal cord injury [12], [13], diabetes [14] and cystitis [15], [16]. These findings not only emphasize the importance of urothelial ATP release and signaling for proper bladder function, but also highlight the need to better understand the cellular mechanisms whereby urothelial cells respond to bladder wall distension with ATP release.

In general, regulated cellular ATP release can occur through vesicular and non-vesicular mechanisms. Vesicular ATP release involves activation of exocytotic mechanisms while non-vesicular ATP release may be mediated by activation of stretch, voltage and/or ligand-gated ion channels and receptors, mitochondrial porins (VDAC), and ATP binding cassette (ABC) transporters [17]. There is evidence that both vesicular and non-vesicular ATP release mechanisms operate in bladder urothelial cells. Several receptors and channels have been shown to participate in these mechanisms, such as the TRPV1 and TRPV4 (Transient receptor potential vanilloid) channels [18]–[23], Piezo1 [24], acid-sensing ion channel (ASIC) [25], epithelial Na^+^ channels (ENaC) [23], [26], muscarinic acetylcholine receptors [27], bradykinin receptors [28], PACAP (pituitary adenylate cyclase-activating polypeptide) PAC_1_ receptor [29] and P2Rs [30], [31]. Observation that removal of extracellular Ca^2+^ augments ATP release from the bladder urothelium [32], a condition known to enhance P2X_7_R activation [33]–[35], strongly suggests the participation of this P2R subtype in mechanisms of urothelial ATP release. In addition, in other cell types P2X_7_R stimulation has been shown to induce ATP release by opening pannexin 1 (Panx1) channels [36]–[38]. Panx1 is a member of the gap junction family of proteins that forms large non-junctional channels which allow diffusion of ions and small molecules (<1 kDa) between the cytosol and extracellular space. Besides being activated by P2X_7_R and other P2Rs, Panx1 channels are sensitive to voltage, high extracellular K^+^ and mechanical stimulation [39]–[41]. Panx1 is expressed in various cell types and has been shown to participate in key cellular events, such as intercellular signaling, mechanotransduction, and inflammatory responses [37], [42]–[47]. The involvement of Panx1 in pathophysiological mechanisms is also becoming increasingly apparent [48]–[54]. We have recently shown that Panx1 contributes to development of neurogenic bladder in mice with experimental autoimmune encephalomyelitis (EAE), a model of Multiple Sclerosis [55]. Panx1 has also been proposed to participate in mechanisms of bladder overactivity involving P2Y_6_R activation [56]. However, little is still known of the actual role played by Panx1 channels in the urinary bladder under physiological conditions.

Based on the characteristic mechanosensitivity of Panx1 channels and their demonstrated function as conduits for cellular ATP release, and the key role of ATP as an urothelial mechanosignaling molecule, in this study we investigated whether Panx1 channels participate in mechanisms of urothelial mechanotransduction and intercellular signaling. First we immunolocalized Panx1 and P2X_7_R in the rat bladder mucosa, and determined the effects of intravesical administration of mefloquine (MFQ, a Panx1 channel blocker) on amounts of ATP released in the bladder lumen in response to bladder distension. Then, to specifically demonstrate the functional interaction of Panx1 and P2X_7_R in the urothelium, we used the TRT-HU1 immortalized human urothelial cell line to measure the effects of pharmacological blockade of Panx1 channels and P2X_7_R on mechanically-induced urothelial ATP release, dye-uptake and transmission of intercellular Ca^2+^ waves, which is a form of long range cell-cell communication mediated by ATP [57]. Bladders and urothelial cells isolated from mice deficient in Panx1 or P2X_7_R were also used in ATP release and dye-uptake experiments to support the pharmacological findings obtained from rat bladders and human urothelial cells. Our findings indicate that Panx1 is expressed in the bladder urothelium, that Panx1 channels provide a mechanosensitive conduit for urothelial ATP release and participate in urothelial ATP signaling by functionally interacting with P2X_7_R. Based on these roles, the Panx1 channel can be viewed as one of the molecular components of the bladder mechanosensory and transduction systems and, as such, is expected to play key roles in the regulation of bladder function.

## Results

### Pannexin 1 channels and P2X_7_ receptors are co-expressed in the rat bladder mucosa

**Figure 1:**
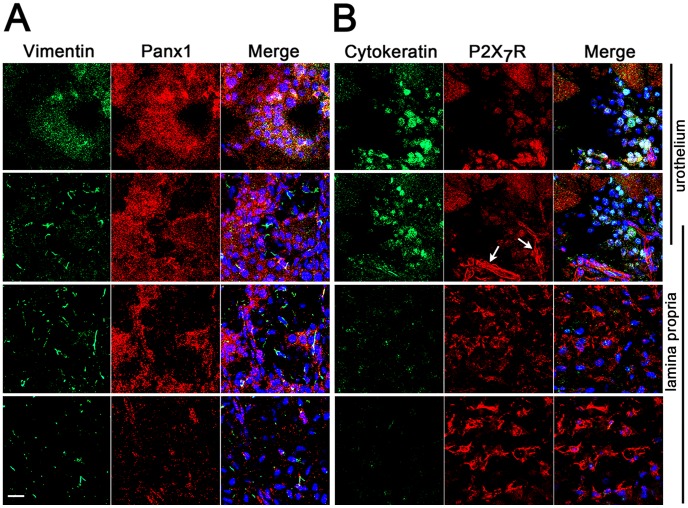
Rat bladder mucosa is immuno-positive for Pannexin 1 (Panx1) channels and P2X_7_ receptors. Confocal fluorescence Z stack images of flat mount bladder mucosa taken from the urothelial towards the serosal surface. (A) Granular staining for Panx1 channels (red) is observed throughout the mucosa. Staining for the intermediate filament vimentin (green) is observed on the apical urothelial region and particularly on a few cells in the lamina propria, which likely correspond to suburothelial myofibroblasts. Note partial colocalization of Pannexin 1 with vimentin-positive cells. (B) Positive staining for P2X_7_R is observed in the urothelium, blood vessels (white arrows) and lamina propria, while staining for cytokeratin 7/17 is restricted to urothelial cells. Note intense P2X_7_R immunoreactivity on the basal region of the mucosa, which is likely localized to the lamina propria myofibroblasts. DAPI nuclear staining in blue. Scale bar = 20 µm.

Immunolabeling and confocal imaging of whole flat mounts of rat bladder mucosa indicated that Panx1 is expressed throughout the urothelium layer and in some parts of the lamina propria (suburothelium) (Figure 1A). In the lamina propria, Panx1 immunostaining partially co-localized with vimentin positive spindle-shaped cells, indicating that some of the suburothelial myofibroblasts also express Panx1 (Figure 1A). Given the acknowledged functional interaction of Panx1 with P2X_7_ receptors (P2X_7_R), where P2X_7_R stimulation have been shown to open Panx1 channels [46], [58], [59], we also immunostained the rat bladder mucosa for these receptors. As described in previous reports [3], [60], [61], P2X_7_R immunoreactivity was observed in the bladder urothelium, lamina propria and blood vessels (Figure 1B). Cells displaying immunoreactivity for P2X_7_R in the suburothelium were spindle-shaped, indicating that myofibroblasts were likely positive for P2X_7_R. This finding of Panx1 and P2X_7_R immunoreactivity throughout the urothelium and in the lamina propria suggests the involvement of Panx1 and P2X_7_R in mucosal function, which led us to analyse its specific role in ATP release.

### Blockade of Pannexin 1 channels reduces stretch-induced ATP release from isolated whole rat bladders

**Figure 2:**
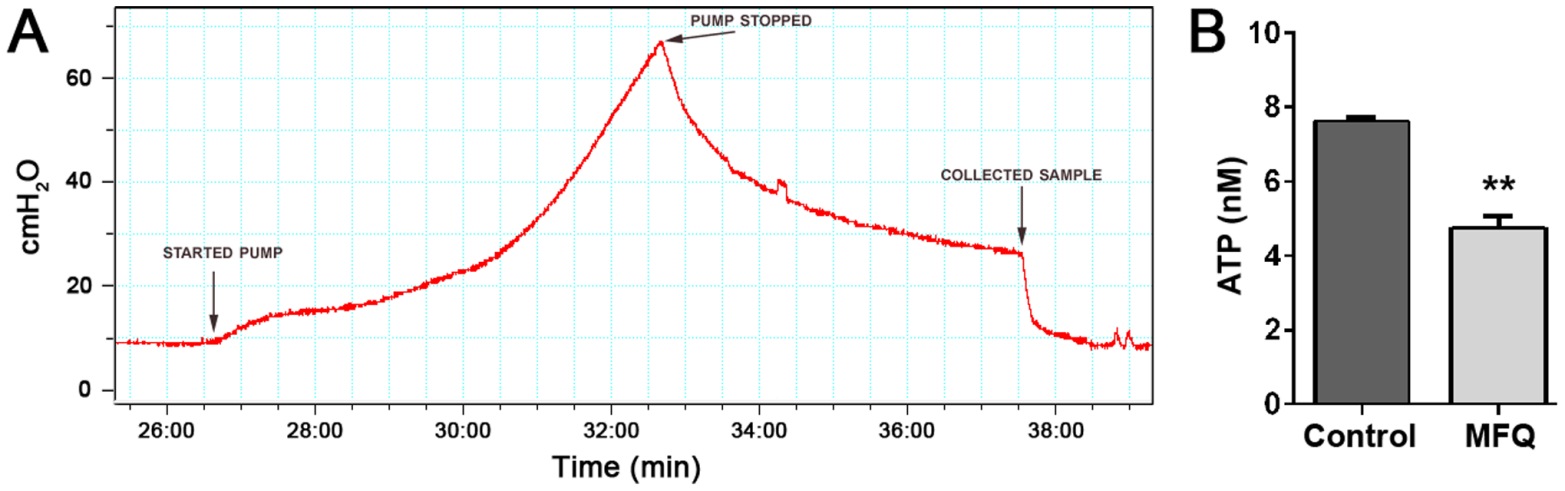
Pannexin 1 channels contribute to mechanically-induced ATP release from bladder mucosa. Whole isolated rat bladders were bathed and instilled with PBS+glucose (1 g/L) in the absence and presence of the Panx1 channel blocker mefloquine (MFQ, 100 nM). A filling-voiding cycle was simulated by bladder instillation for 6 min at 10 mL/h flow rate followed by 5 min no flow, after which the bladder was emptied and ATP release in the bladder lumen was quantified. (A) Representative recording of intravesical pressure before, during instillation and at the moment of sample collection (“voiding”). (B) Bladder wall distension induced release of significant amounts of ATP that was blunted in the presence of 100 nM MFQ. Data represent mean±SEM (N = 3 bladders per experimental condition. **P<0.01 by Student’s *t*-test).

Evidence for the functional expression of Panx1 channels in the urinary bladder was first obtained using mefloquine (MFQ). Panx1 channels are highly sensitive to blockade with low concentrations of MFQ (≤100 nM) [62], and pharmacological approaches using this synthetic quinine analogue have thus been broadly used to indicate and study the involvement of these channels in physiological and pathological events. The effects of MFQ on ATP release from isolated whole rat bladders was investigated using an intravesical perfusion and pressure monitoring setup that allows simulation of the mechanical distension that the bladder wall sustains during a normal urine filling cycle. As shown in figure 2A, pressure-induced bladder distension resulted in ATP release into the bladder lumen that was significantly reduced by adding MFQ (100 nM) in the instillation solution (Figure 2B).

### Absence of pannexin 1 channels and P2X_7_ receptors blunts stretch-induced ATP release from isolated whole mouse bladders

**Figure 3:**
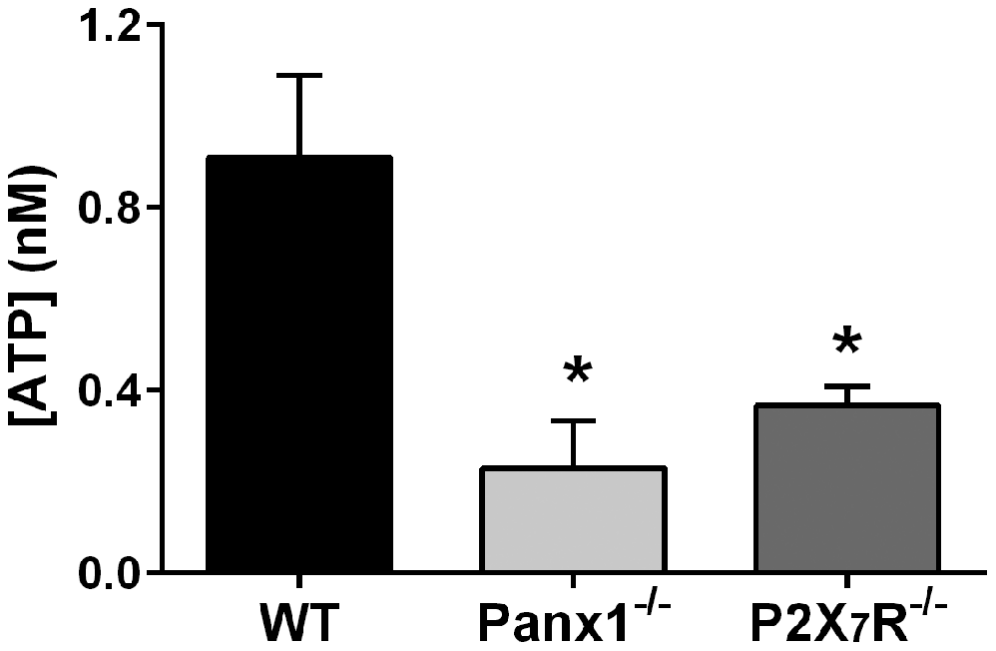
Stretch-induced ATP release is reduced in bladders of pannexin 1 and P2X_7_ receptor deficient mice. Whole bladders isolated from wildtype (WT), Panx1 deficient (Panx1^−/−^) and P2X_7_R deficient (P2X_7_R^−/−^) mice were bathed and instilled with PBS+glucose (1 g/L). A filling-voiding cycle was simulated by bladder instillation for 8 min at 1.5 mL/h flow rate followed by 5 min no flow, after which the bladder was emptied and ATP release in the bladder lumen was quantified. Data represent mean ± SEM (N = 9 WT, 4 Panx1^−/−^ and 7 P2X_7_R^−/−^ bladders. Compared to WT: *P<0.05 and **P<0.01 by Student’s *t*-test).

Although use of pharmacological approaches has been instrumental in the identification and investigation of the participation of particular molecular mediators and signaling pathways in physiological and pathological mechanisms, there is always a concern regarding drug-target selectivity. In this experiment we thus used bladders from mice deficient in Panx1 or P2X_7_R to further demonstrate the involvement of Panx1 channels in mechanisms of stretch-induced ATP release and to investigate whether P2X_7_R also participates in these mechanisms, respectively. As shown in figure 3, luminal ATP release in response to mechanical distension was significantly lower in Panx1^−/−^ when compared to that measured from wildtype bladders (Fig. 3). This finding confirms those obtained from rat bladders using the Panx1 channel blocker MFQ. In addition, besides further indicating the involvement of Panx1 channels in stretch-induced ATP release, they also demonstrate the adequacy of using MFQ as a pharmacological tool in future studies with rat or mouse models aimed at investigating the contribution of Panx1 in bladder pathophysiological mechanisms. Distension-induced ATP release was also significantly lower in P2X_7_R^−/−^ bladders compared to wildtype bladders (Fig. 3), which indicates the involvement of this P2R subtype in mechanisms of stretch-induced luminal ATP release. Activation of P2X_7_R has been shown to mediate ATP release through a mechanism that involves opening of Panx1 channels [37], [38], [45], [63]. To further investigate the role of Panx1 and of its interplay with P2X_7_R in the bladder mechanotransduction and signaling systems, specifically focusing on the bladder urothelium, we performed *in vitro* studies with cultured urothelial cells.

### Pannexin 1 channels and P2X_7_ receptors are expressed in human urothelial cells

**Figure 4:**
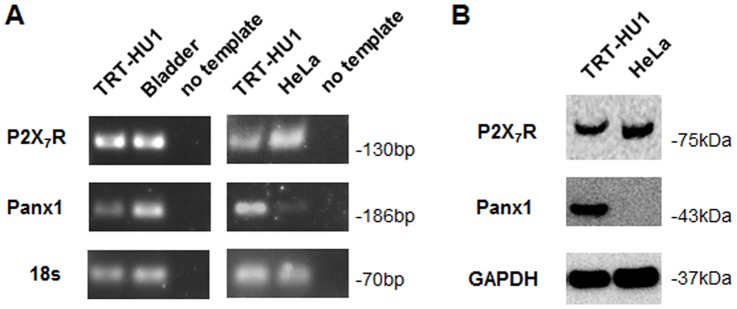
Immortalized human bladder urothelial (TRT-HU1) cells express Panx1 and P2X_7_R. Detection of Panx1 and P2X_7_R mRNA by PCR in (A) and protein by immunoblotting in (B). Total RNA from human bladder and HeLa cells were used as reference for the PCR analyses, and whole HeLa cell lysates was used as reference for immunoblotting.

An urothelial cell line instead of primary urothelial cell cultures was used to allow long term culturing and thereby formation of monolayers with larger number of cells, which was essential for the ATP release and intercellular signaling experiments conducted in this study. The adequacy of our hTERT-immortalized human urothelial cell line (TRT-HU1) for the proposed studies was first demonstrated by co-expression of Panx1 and P2X_7_R in these cells at both mRNA and protein level, as illustrated in figure 4. Expression of *PANX1* and *P2RX7* mRNA was detected by RT-PCR, and human bladder and HeLa cells were used as references (Figure 4A). Expression of Panx1 and P2X_7_R in TRT-HU1 cells at the protein level was confirmed by immunoblotting and HeLa cells were used as reference (Figure 4B).

### Pannexin 1 channels are functionally expressed in TRT-HU1 cells and functionally interact with P2X_7_ receptors

**Figure 5:**
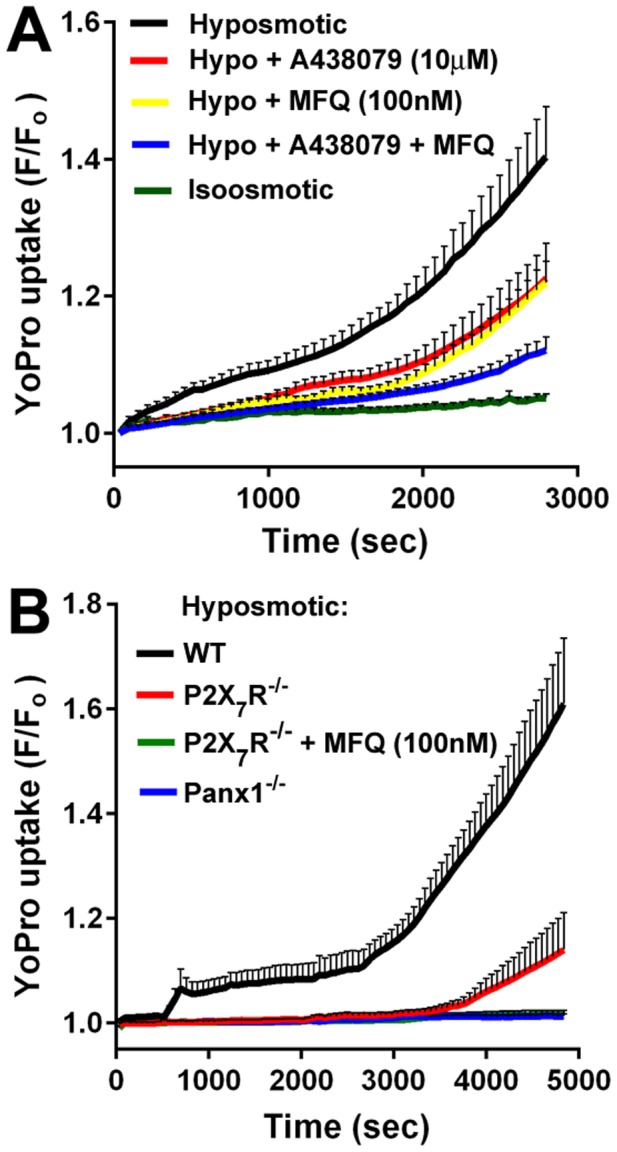
YoPro-1 uptake by urothelial cells in response to hypoosmotic shock involves Panx1 and P2X_7_R activation. (A) TRT-HU1 cells: YoPro-1 uptake induced by cell swelling (hypoosmotic shock, black line) was significantly reduced in the presence of the Panx1 channel blocker mefloquine (MFQ 100 nM; yellow line), the P2X_7_R blocker A438079 (10 µM; red line) and when both Panx1 and P2X_7_R were blocked (blue line). Except for hypoosmotic with MFQ vs. A438079 and hypoosmotic with A438079+MFQ vs. isoosmotic, all other comparisons were significantly different at time 2800 sec (*P*<0.01, N≥6) by two-way repeated measures ANOVA, followed by Tukey’s multiple comparison. (B) Primary mouse urothelial cells: YoPro-1 uptake induced by hypoosmotic shock was significantly lower in P2X_7_R^−/−^ (red line) compared to wildtype (WT) urothelial cells (black line). Dye uptake by P2X_7_R^−/−^ cells was abolished in the presence of the Panx1 channel blocker mefloquine (MFQ, 100 nM; green line) and was absent in Panx1^−/−^ urothelial cells (blue line). WT vs P2X_7_R^−/−^, P2X_7_R^−/−^+MFQ and Panx1^−/−^ (*P<*0.001, N = 4) by ANOVA followed by Tukey’s multiple comparison.

Functional expression of Panx1 channels in TRT-HU1 cells was evaluated based on the unique characteristics of these channels. Panx1 channels form large pores that allow diffusion of ions and small molecules (<1 kDa) between the intracellular and extracellular compartments. Dye uptake assays using large molecular weight dyes, such as YoPro-1 (629 Da) whose fluorescence is enhanced when it binds to nucleic acids, are commonly used to detect opening of Panx1 channels and other large permeation pores. Thus, to investigate whether Panx1 channels in TRT-HU1 cells were functional and sensitive to mechanical stretch we submitted the cells to 50% hypoosmotic shock in the presence of YoPro-1. As shown in figure 5A, hypoosmotic shock induced significant YoPro uptake in TRT-HU1 cells and this response was significantly reduced in the presence of MFQ (100 nM). Dye-uptake by TRT-HU1 cells was also significantly reduced by A438079 (10 µM) (Figure 5A), a P2X_7_R antagonist [64]. When combined, MFQ and A438079 displayed synergistic inhibitory effects on dye-uptake by TRT-HU1 cells, which indicates that both Panx1 and P2X_7_R participate in this event. Inhibition of YoPro dye-uptake by A438079 might be attributable to unspecific effects on Panx1 channels [65], [66]. To definitely demonstrate the involvement of P2X_7_R in this response, we established short-term primary cultures of urothelial cells isolated from P2X_7_R^−/−^ and from Panx1^−/−^ mouse bladders. As shown in figure 5B, dye-uptake was significantly lower in P2X_7_R^−/−^ compared to wildtype urothelial cells, and was completely inhibited when P2X_7_R^−/−^ cells were treated with the Panx1 channel blocker MFQ (Figure 5B). Absence of Panx1 had more marked effects and completely prevented dye-uptake (Figure 5B). These observations corroborate the pharmacological findings with TRT-HU1 cells and further indicate the participation of both Panx1 and P2X_7_R. However, findings that Panx1 absence completely abolished dye uptake, whereas P2X_7_R only reduced it, indicates that P2X_7_R participation in this event requires Panx1 presence.

### Pannexin 1 channels and P2X_7_ receptors contribute to mechanically-induced ATP release

**Figure 6:**
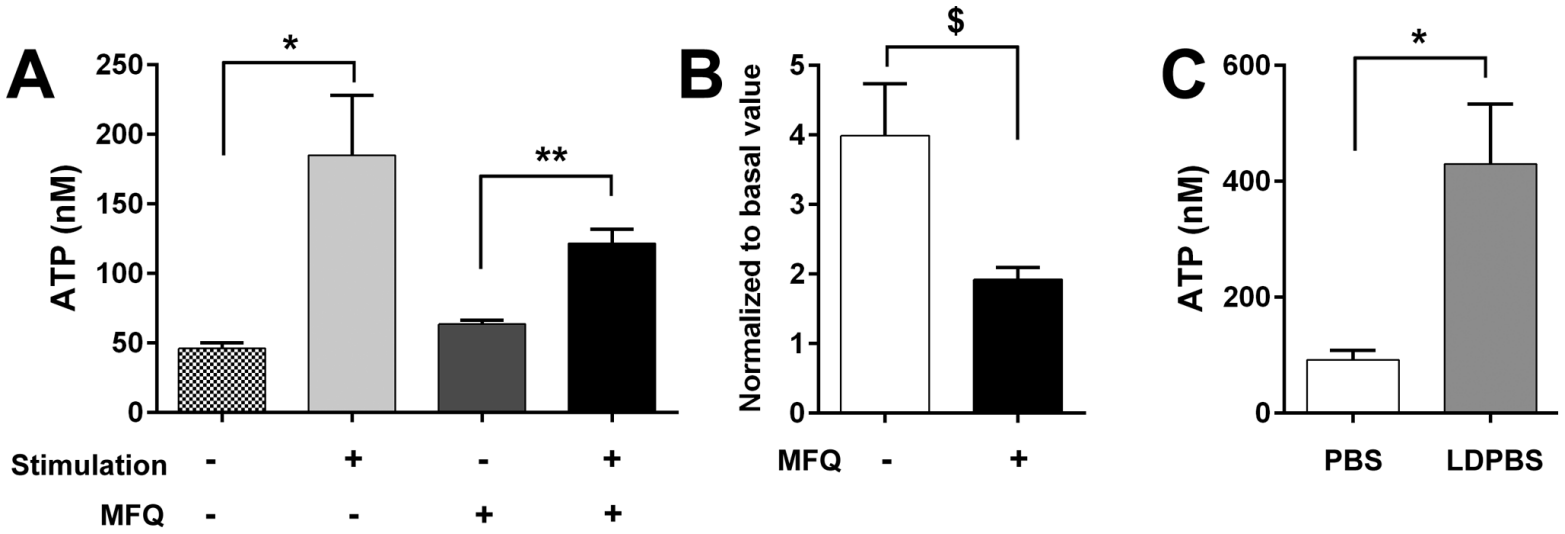
ATP release is reduced by Panx1 channel blockade and enhanced by low divalent cation solution. (A) Mechanical stimulation imposed by rinsing the cells with bathing solution induced ATP release from TRT-HU1 cells that was significantly higher than that from non-stimulated cells when measured in the presence or absence of the Panx1 channel blocker mefloquine (MFQ, 100 nM; n = 4 each, *P<0.05 and **P<0.01, by paired *t*-test.). (B) Normalized ATP release with respect to basal values, however, was significantly lower in MFQ-treated compared to untreated cells (N = 4, ^$^ *P*<0.05 by Mann-Whitney U test). (C) Exposure to low divalent cation solution (LDPBS), a condition known to enhance P2X_7_R activation, significantly increased ATP release from TRT-HU1 cells. All data represent mean ± SEM (N = 4 each, *P<0.05 by Student’s *t*-test).

Evidence that Panx1 channels provide the mechanosensitive conduit for cellular ATP efflux from bladder urothelium was obtained by investigating the effects of MFQ on ATP release from TRT-HU1 cells. Mechanical stimulation imposed by rinsing with cell bathing solution induced significant ATP release in the presence and absence of MFQ (Figure 6A). However, mechanically-induced ATP release (normalized with respect to basal values) was significantly lower (∼2-fold) in MFQ-treated compared to control untreated cells (Figure 6B). Evidence of P2X_7_R activation following mechanical stimulation and of its involvement in mechanically-induced ATP release from urothelial cells was obtained from experiments where cells were bathed in low divalent cation PBS (LDPBS). Exposure to low divalent cation enhances P2XR activation but, most notably, that of P2X_7_R [33]–[35], [67], which is clearly expressed in TRT-HU1 cells (Figure 4). As shown in figure 6C, ATP release from TRT-HU1 cells was significantly increased in LDPBS.

Findings from these experiments combined with those from stretch-induced luminal ATP release, mechanically-induced dye uptake and functional co-expression of Panx1 and P2X_7_R indicate that Panx1 channels participate in urothelial mechanotransduction mechanisms by providing a mechanosensitive and a P2X_7_R-sensitive conduit for cellular ATP efflux.

### Pannexin 1 channels and P2X_7_ receptors participate in mechanisms of ATP signaling between urothelial cells

**Figure 7:**
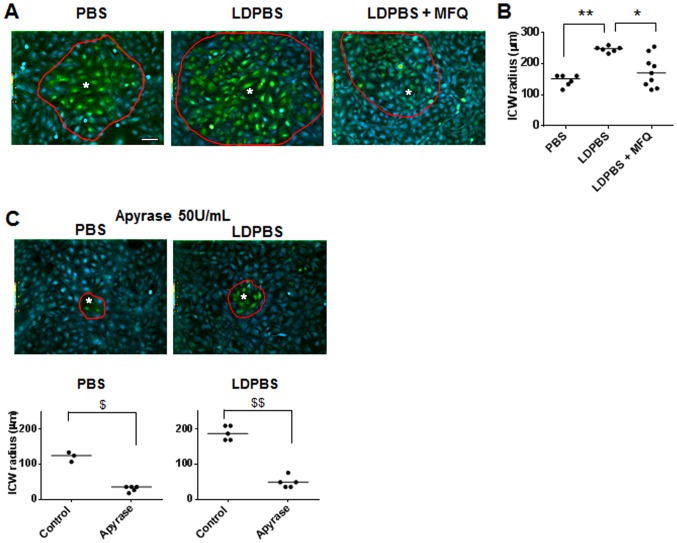
Pannexin 1 channels and P2X_7_Rs participate in ATP-mediated paracrine signaling among urothelial cells. Intercellular communication in TRT-HU1 cultures was induced by focal mechanical stimulation of single cells (white asterisks) and measured as radius of the intercellular Ca^2+^ wave (ICW) spread. (A) Representative pseudocolor images illustrating the radius (indicated by a red line circle) of ICW spread in PBS, in low divalent cation solution (LDPBS) and in LDPBS+mefloquine (MFQ, 100 nM). (B) Amplification of ICW spread in LDPBS is prevented by treatment with the Panx1 channel blocker MFQ (N = 6, 6 and 9, respectively. *P<0.05 and **P<0.01 by Kruskal-Wallis test). (C) Apyrase, an ATP catalase, greatly reduced the radius of ICW spread in TRT-HU1 cultures bathed in both PBS (N = 3 and 5, ^$^ *P*<0.05 by Mann-Whitney U test) and LDPBS (N = 5 and 6, ^$$^ *P*<0.01 by Mann-Whitney U test). Bar: 100 µm.

The role of urothelial-derived ATP as a transmitter in the communication of bladder distension to the CNS is well recognized. Little is still known, however, of the role played by distension-induced ATP signaling within the urothelium. In several cell types, ATP released in response to mechanical, chemical and/or electrical stimulation can induce increase in intracellular Ca^2+^ level of the stimulated cells and trigger transmission of intercellular Ca^2+^ waves (ICWs), a form of long range signaling believed to be important for coordination of cellular activity [57]. ICW transmission is supported by a paracrine pathway mediated by ATP release and activation of P2Rs, and a gap junction-mediated pathway that allows cytosol-to-cytosol diffusion of Ca^2+^-mobilizing second messengers such as Ca^2+^ and IP_3_ between neighboring coupled cells [57], [68]. TRT-HU1 cells express P2Rs (Figure 4 and Figure S1) and are coupled by gap junctions [55]. These cells are thus fully equipped to communicate through transmission of ICWs. As expected and shown in figure 7, focal mechanical stimulation of single cells in confluent TRT-HU1 cell cultures triggered ICWs that reached cells located far apart from the stimulated cells. The extent to which the paracrine ATP-mediated pathway contribute to signaling between TRT-HU1 cells is demonstrated by adding apyrase, an ATP scavenger, in the cell bathing solution. In the presence of apyrase (50 U/ml) the transmission of Ca^2+^ signals was confined to cells in the immediate neighborhood of the mechanically-stimulated cells (Figure 7C). We have shown that activation of Panx1 and P2X_7_R in astrocyte cultures contributes to ICW transmission and modulates the range of ICW spread by providing a feed-forward mechanism of ATP-induced ATP release [37], [38], [45], [63]. To specifically investigate the involvement of Panx1 and P2X_7_R in ICW transmission we exposed the cells to LDPBS and MFQ. As shown in figure 7A, the radius of the ICWs spread in TRT-HU1 cultures was significantly increased when cells were bathed in LDPBS, a response that was markedly reduced by MFQ (100 nM). Evidence that this increase in range of Ca^2+^ signaling between TRT-HU1 cells is related to an enhanced ATP release from stimulated cells was provided by the observation that apyrase also abrogated ICW spread in LDPS but to a lesser extent than observed in PBS (Figure 7C). Overall, these findings of intercellular Ca^2+^ signaling amplification in low divalent cation conditions and its sensitivity to MFQ and apyrase supports the role of ATP as the extracellular messenger and the participation of P2X_7_R and Panx1 channels in mechanisms of distension-induced ATP release and urothelial signaling.

## Discussion

Our view of the role played by the urothelium in bladder function changed radically over the last fifteen years since the demonstration by Ferguson and colleagues that distension of the bladder wall, as occurs during bladder filling with urine, induces release of significant amounts of ATP from the urothelium [32]. This finding led to the proposal that besides acting as a selective barrier that separates and protects the bladder from the urine contents [69], the urothelium also functions as a sensor for changes in intravesical pressure [32], [70]. Several studies have since been conducted to identify the molecular mediators and mechanisms involved in urothelial mechanotransduction and ATP release. In this study we provide evidence that the Panx1 channel is one of these molecular mediators. We show that Panx1 channels are expressed throughout the bladder mucosa and in TRT-HU1 immortalized human urothelial cells, and that ATP release in response to bladder wall distension and mechanical stimulation of TRT-HU1 cells is inhibited by the Panx1 channel blocker mefloquine (MFQ) and is blunted in Panx1 deficient mice. The characteristic mechanosensitivity and the large size and permeability of the pore formed by the Panx1 channel [39]–[42] make this channel an ideal candidate for a role in the urothelial mechanosensory and transduction systems. In other cells that are also naturally subjected to mechanical stimulation, such as erythrocytes [71], airway epithelial cells [72], [73] and bone cells [43], Panx1 channels have also been shown to provide a mechanosensitive pathway for ATP release and dye-uptake.

Besides responding to cell surface deformation, Panx1 channels can also be activated by cellular depolarization, increase in intracellular Ca^2+^ and extracellular K^+^ and have been shown to be the “large permeation pore” recruited by P2X_7_R activation [46], [59]. The precise mechanisms whereby P2X_7_R activates the Panx1 channel are still unknown, but there is evidence that a tyrosine kinase of the Src family participates in the initial events leading to Panx1 channel opening following P2X_7_R stimulation [38], [58]. This sensitivity of Panx1 channels to P2X_7_R stimulation creates a peculiar situation in which activation of either one can result in the activation or enhanced activation of the other, triggering a cycle of reciprocal activation where ATP release induces further ATP release. Such a mechanism may have dire consequences and lead to cell death when it is not controlled. In this regard, observations that extracellular ATP can inhibit Panx1 channels suggest that an autoregulatory mechanism may modulate P2X_7_R-Panx1 activation and control ATP-induced ATP release [65]. The relevance of this functional interplay between Panx1 channels and P2X_7_R is becoming increasingly apparent. For example, activation of the P2X_7_R-Panx1 complex has been proposed to modulate the range of intercellular signaling in the astrocytic network [45], [63], has been implicated in processing and release of interleukin-1β [46], and to mediate inflammation-induced enteric neuron death [48]. This functional interplay between P2X_7_R and Panx1 seems to be broadly observed. However, exceptions may also exist, as reported for macrophages [74], in which another still unknown membrane protein has been proposed to be involved in P2X_7_R pore formation [75].

In this study, using both pharmacological and genetic approaches, we demonstrate that Panx1 channels and P2X_7_R are functionally co-expressed in urothelial cells. This functional interplay between P2X_7_R and Panx1 is clearly observed here in the YoPro uptake, ATP release and ICW spread experiments performed with TRT-HU1 cells. Most notable, however, are our findings that Panx1 channels not only provide the distension-induced ATP release that can initiate paracrine signaling between urothelial cells, but may also provide ATP release from neighboring non-stimulated cells through P2X_7_R stimulation. In this scenario, ATP-induced ATP release mediated by activation of the P2X_7_R-Panx1 complex would not only support the mechanically-initiated intercellular signaling among urothelial cells but provide a mechanism for mechanosensory amplification. A role for urothelial-derived ATP as the transmitter that communicates bladder distension to the CNS through activation of P2Rs in suburothelial afferent nerve terminals is broadly accepted. However, the actual role played by distension-induced ATP mediated signaling within the urothelium is still largely unknown, but is expected to be important for urothelial function as a syncytium, providing for synchronization and coordination of the urothelial cells. For example, activation of P2Rs has been shown to be essential for increasing the apical surface area of the urothelium during bladder filling [76]. In this regard, ATP release and signaling within the same and between urothelial layers is likely essential to convey the information of bladder distension and provide for proper synchronization of urothelial cell response and adaptation to bladder wall distension.

In this study we focused on investigating the role of Panx1 channels and P2X_7_Rs in urothelial ATP release and signaling. Our immunohistochemical studies, however, indicate that in the rat bladder mucosa Panx1 channels and PX_7_R are also expressed by spindle-shape cells in the lamina propria that likely correspond to suburothelial myofibroblasts. These cells are in close contact with suburothelial nerves [77] and form a network functionally connected by gap junctions [78]. Isolated suburothelial myofibroblasts have been shown to respond to exogenous ATP with generation of intracellular Ca^2+^ transients [79], [80] and when mechanically stimulated in intact bladder cross-sections they initiate transmission of ICWs, which spread across the suburothelial network and invade the underlying detrusor layer [81]. These features prompted the proposal that suburothelial myofibroblasts may act as amplifiers in the sensory response to bladder wall distension [81], [82]. Future studies are needed to determine whether Panx1 channels are functionally expressed in suburothelial myofibroblasts. Given the characteristic properties of Panx1 channels and findings presented here for urothelial cells, we can speculate that Panx1 channels and P2X_7_R may also participate in responses of suburothelial myofibroblasts to mechanical stimulation and in ATP signaling among these cells. Similar to its role discussed here for the urothelium, the P2X_7_R-Panx1 complex could be a key participant in a mechanism for mechanosensory amplification at the level of the suburothelial layer and signaling of bladder wall distension from the bladder mucosa to the CNS and detrusor smooth muscle cells. Perception of bladder distension is essential for proper micturition function and the role played by urothelial ATP signaling in the bladder mechanosensory and transduction pathways is indisputable. Our demonstration that Panx1 channels are among the several types of receptors and channels that participate in mechanisms of urothelial ATP release adds to the complexity of the urothelial mechanosensory and transduction apparatus and significantly advances our understanding of the bladder intrinsic regulatory mechanisms.

## Methods

### Ethics Statement

Animals were treated in strict accordance with the National Institutes of Health (NIH) animal care guidelines and experimental procedures were approved by the Einstein Animal Care and Use Committee (IACUC approval numbers: 20110307 and 20110308).

### Animals

Sprague Dawley female rats (250–300 g) were purchased from Harlan Laboratories (Indianapolis, IN, USA). Panx1 deficient (Panx1^−/−^) on the C57Bl/6 background were generated in our animal facility at the Albert Einstein College of Medicine (Einstein) by breeding heterozygous Panx1^tm1a(KOMP)Wtsi^ purchased from the Knockout Mouse Project (KOMP) at UCDavis [83]. P2X_7_R deficient mice (P2X_7_R^−/−^) [84] were purchased from Jackson Laboratories (B6.129P2-*P2rx7* ^tm1Gab^/J). Homozygous Panx1^+/+^ mice from our breeding colony and C57Bl/6 mice purchased from Jackson laboratories were used as controls in experiments with Panx1^−/−^ and P2X_7_R mice, respectively. All animals were housed in the Institute of Animal Care and Use Committee approved animal facilities at Einstein. Food and water were available ad libitum.

### Controlled pressure-induced bladder distension

Animals were sacrificed in a CO_2_ chamber, the urinary bladders dissected and placed in phosphate buffered saline (PBS, Mediatech, Manassas, VA) containing 1 g/L glucose (PBS+G). The bladders were then suspended in a water-jacketed reservoir (36°C) attached by the neck to a pressure and instillation setup and bathed in aerated PBS+G. The setup was coupled to a disposable pressure transducer (Deltran, Utah Medical Products, Midvale, UT, USA) and a syringe infusion pump (model SP101i, World Precision Instruments, Sarasota, FL, USA) to allow for bladder instillation (when in the open configuration) and for controlled increase in intravesical pressure/bladder distension (when in the closed configuration). Bladders were instilled for 15 min with PBS+G (flow rate: rat = 1.5 mL/h and mouse = 0.6 mL/h; setup in the open configuration) to rinse the bladder lumen, followed by a 10 min rest period at basal intravesical pressure of 5–10 cm H_2_O (setup in the closed configuration). After this equilibration period and still in the closed configuration, the rat and mouse bladders were instilled at a rate of 10 and 1.5 mL/h [routinely used in rat [85] and mouse [86] cystometry, respectively] for 6 and 8 min [final intravesical volume ∼0.8 and 0.2 mL, based on average voided volume per micturition measured from rats [85] and mice [55], respectively]. The pump was then stopped and 5 min after stopping the pump the bladder was emptied and the intravesical solution frozen in liquid N_2_ for subsequent ATP measurement. Changes in intravesical pressure were recorded throughout the procedure on an ADI system (PowerLab 4/20 with Chart 5, ADInstruments, Colorado Springs, CO, USA). Experiments were performed in the absence and presence of the Panx1 channel blocker mefloquine (MFQ, 100 nM; QU024-1, BioBlocks, San Diego, CA), which was maintained throughout the experiment diluted in the instillation and in the bathing PBS+G solutions.

### Immunohistochemistry

Rat bladders were isolated as described above, placed in cold PBS, opened longitudinally and cut in four strips. The bladder mucosa from each strip was then mechanically separated from the detrusor muscle using fine tweezers and a dissecting microscope, and fixed with 4% paraformaldehyde (PFA) at 4°C for 24 h. The tissues were then washed with PBS (3 times, 5 min) and bathed for 15 min in 50 mM ammonium chloride (in PBS) to remove cross-linking induced by PFA fixation. After washing again with PBS (3 times, 5 min), the tissue was permeabilized for 30 min with 0.25% Triton X-100 (in PBS), blocked for 60 min with 1% bovine serum albumin (BSA, in PBS) and incubated 3 days with primary antibodies (in 1% BSA +0.4% Triton X-100). The mucosa strips were then washed with PBS (3 times, 15 min) and incubated for 2 h at room temperature with secondary antibodies (in PBS +1% BSA). After rinsing with PBS (3 times, 15 min) the mucosa strips were individually placed on *Superfrost Plus* microscope slides, gently cut in half and one of the halves was flipped over so as to have the urothelium facing either up or down. The tissue was then flat mounted with square glass coverslips (22×22 mm) in a glycerol based mounting media containing 0.2% n-propyl gallate and 4 mM DAPI (Sigma-Aldrich, St. Louis, MO). The following primary antibodies were used: affinity purified polyclonal rabbit anti-rat P2X_7_R corresponding to amino acid residues 576–595 (1∶250, Alomone Labs, Jerusalem, Israel), polyclonal rabbit anti-mouse Pannexin 1 CL (Cytoplasmic loop, 1∶50, Invitrogen, Carlsbad, CA), monoclonal mouse anti-human cytokeratin 7/17 (1∶100, Santa Cruz Biotechnology, Dallas, TX) and monoclonal mouse anti-vimentin (1∶100, Sigma-Aldrich). Secondary antibodies: Alexa Fluor 488 donkey anti-mouse IgG (H+L) and Alexa Fluor 594 donkey anti-rabbit IgG (H+L), both from Invitrogen.

### Confocal microscopy

Immuno-positive images for Pannexin 1 channels, P2X_7_ receptors, vimentin and cytokeratin7/17 in the bladder mucosa flat mounts was acquired with Zeiss LSM 510 DUO Laser Scanning Confocal Microscope (Carl Zeiss, Germany) using a 40× water-immersion objective. Images were taken serially from the urothelial apical surface towards the basal region of the mucosa at 0.6 µm z axis steps. Total thickness of the flat mounted mucosa strips was 20 to 50 µm.

### Cell culture

The hTERT-immortalized human urothelial cell line (TRT-HU1) [87] was a gift from Dr. Rosalyn M. Adam (Urological Diseases Research Center, Children’s Hospital Boston, Boston). TRT-HU1 cells were maintained in Dulbecco’s Modified Eagle Medium (DMEM, GIBCO, Life Technologies, Grand Island, NY) containing 2 mM L-glutamine and 110 mg/L sodium pyruvate supplemented with 15% fetal bovine serum (FBS, GIBCO), non-essential amino acids (GIBCO), and 1.15 mM 1-thioglycerol, as previously described [87]. Primary mouse urothelial cells were prepared following a protocol modified from Kullmann et al. [27]. Briefly, mice were deeply anesthetized with isoflurane and euthanized by cervical dislocation followed by decapitation. Bladders were removed, placed in cold minimal essential medium (MEM; Invitrogen, Life Technologies) supplemented with HEPES (2.5 g/l; Sigma-Aldrich) and 1% penicillin/streptomycin (Invitrogen, Life Technologies), and cut longitudinally in four strips. The strips were then incubated in dispase (1.0 mg/ml; Stemcell Technologies, Vancouver, Canada) for 2 hours at 36°C. Next, the strips were transferred to warm MEM containing 10% FBS, urothelial cells were gently scraped from the underlying tissue and centrifuged at 1000 g for 10 min. The cells were resuspended in keratinocyte media (GIBCO) and plated on ibid µ-Dish 35 mm tissue culture treated dishes (ibidi, Verona, WI). Cells were used 24 h after dissociation.

### RT–PCR

Total RNA was extracted from TRT-HU1 and HeLa cells using the RNeasy plus mini kit (Qiagen, Valencia, CA) and complementary DNA was synthesized from 1 µg of RNA using Superscript VILO cDNA Synthesis Kit (Invitrogen, Life Technologies). Primers were designed using Primer Express 2.0 software (Applied Biosystems, Life Technologies). The primers were as follows: P2X_7_, Fwd: AAAGGAATTCAGACCGGAAGG Rev: AGTTTTCGGCACTGTTCAAGAG, Panx1, Fwd: GGCAGAGCTCCAAGGTATGAA Rev: GCAAACCAGCTGTGAAACCA and 18 s ribosomal RNA, Fwd: CACGGCCGGTACAGTGAAAC Rev: AGAGGAGCGAGCGACCAAA. Reaction mixtures were denatured at 95°C for 10 min, followed by 20–40 PCR cycles (PTC-100 Thermal Cycler, MJ Research Inc, Waltham, MA). Each cycle consisted of following three steps: 94°C for 15 s, 57°C for 15 s, and 72°C for 1 min. Human bladder RNA was purchased from Invitrogen.

### Immunoblotting

Whole cell lysates from TRT-HU1 and HeLa cells were prepared in sample buffer (1 mM NaHCO_3_, 2 mM phenylmethylsulphonyl fluoride, 1 mM Na_3_VO_4_, 5 mM EDTA, 1% Triton X-100) containing proteinase inhibitors (Roche laboratories, Basle, Switzerland). Samples with equal protein concentration were resolved by SDS-PAGE and transferred to a nitrocellulose membrane (Whatman, Dassel, Germany). After 30 min incubation with blocking buffer containing 0.5% Tween-20 (TBS-T) and 5% nonfat dry milk, at room temperature, the membranes were incubated overnight at 4°C with polyclonal rabbit anti-human P2X_7_R corresponding to amino acid residues 103–358 (1∶1000, EPITOMICS, Burlingame, CA, USA), polyclonal rabbit anti-mouse Pannexin 1 (N-terminal, 1∶100, Invitrogen), monoclonal GAPDH antibody (1∶20,000 Fitzgerald, Acton, MA, USA). The immunoreactivities were visualized with enhanced chemiluminescence HRP-conjugated anti-rabbit/mouse IgG antibody.

### Dye uptake

The cell impermeant green-fluorescent dye YoPro-1 iodine (629 Da; Molecular Probes, Life Technologies), which exhibits an 1800-fold fluorescence enhancement after binding to nucleic acids, was used in this experiment. TRT-HU1 cells plated on glass bottom MatTek dishes (Ashland, MA, USA) were bathed for 5 min in Krebs-Henseleit containing YoPro-1 (5 µM) and basal dye-uptake was measured. The cells were then submitted to mechanical stimulation by exposure to 50% hypoosmotic shock and YoPro-1 fluorescence was captured continuously for 30 min. Experiments were performed in the absence and presence of the Panx1 channel blocker mefloquine (MFQ, 100 nM) and/or the P2X_7_R antagonist A438079 (TOCRIS bioscience, 10 µM). YoPro-1 fluorescence was measured from regions of interest placed on cells using the MetaFluor software (Molecular Devices, Sunnyvale, CA) and an Orca-ER CCD camera (Hamamatsu Photonics, Hamamatsu, Japan) coupled to an inverted Nikon microscope (Eclipse TE-2000, Japan) equipped with a 20× dry objective and FITC filter set.

### Measurement of ATP release

The Luciferin-luciferase assay (ATP Determination kit, Molecular Probes) was used to quantify amounts of ATP released in the bladder lumen and in the bathing solution of TRT-HU1 cells in response to rinsing-induced mechanical stimulation. Briefly, 5 µl of the collected intravesical or the bathing solution samples, and PBS+G or LDPB+G (for background correction) were individually placed in triplicates in white walled 96-well plates. Immediately after starting the reaction by adding 50 µl of a buffered solution containing luciferin (50 µM) and luciferase (1.25 µg/ml) to each well, the plate was transferred to the FLUOStar plate reader (BMG Labtech, Ortenberg, Germany) and luminescence measured using a 5 sec integration time. The ATP concentration in the samples was calculated from standard curves constructed using ATP from 50 nM to 5000 nM.

### Intercellular calcium waves

TRT-HU1 cells plated on glass bottom MatTek dishes were loaded with the ratiometric intracellular Ca^2+^ indicator Fura-2 AM (10 µM; Molecular Probes) for 45 min at 37°C. Cells were then washed with PBS+G and imaged on a Nikon inverted microscope (Eclipse TE2000-U; Nikon, Tokyo, Japan) equipped with a CCD digital camera (Photometrics CoolSnap HQ2, Tucson, AZ, USA) and a 10× objective (Nikon). Changes in Fura-2 fluorescence intensities emitted at two excitation wavelengths (340 nm and 380 nm) were acquired at 1.0 Hz using a Lambda DG-4 filter changer (Sutter Instruments, Burlingame, CA, USA) driven by a computer through MetaFluor software. Intercellular calcium waves (ICWs) were triggered by focal mechanical stimulation of single cells in the center of the microscope field of view using a glass microelectrode, as previously described [63]. Transmission of ICWs was analyzed in terms of the radius of the Ca^2+^ signal spread measured from concentric tiers (∼40 µm per tier) set around the stimulated cell. ICW spread was measured before and after exposure to LDPBS (low-divalent PBS; containing nominally zero Ca^2+^ and 43 µM MgCl_2_, prepared by dissolving 1 mM MgCl_2_ and 1 mM EDTA in the Ca^2+^- and Mg^2+^-free PBS), as previously described [63], and in the presence and absence of mefloquine (MFQ, 100 nM) and/or apyrase (50U/mL). Values of intracellular Ca^2+^ levels determined from regions of interest placed on cells were obtained from Fura-2 ratio images using an *in vitro* calibration curve, as previously described [63].

### Statistical analysis

For two-group comparison, we used paired or unpaired Student *t*-test for parametric comparison and Mann-Whitney U test for non-parametric comparison. Two-way repeated measures ANOVA followed by Tukey’s multiple comparison was used to compare differences among all experimental groups in the YoPro dye uptake assay. For the ICW experiments, data was analysed using Kruskal Wallis test followed by Dunn’s *post-hoc* test to compare three groups. All statistical analyses were performed using the Graphpad Prism5 (Graphpad Software Inc., San Diego, CA).

## Supporting Information

Figure S1 **Representative immunoblots showing expression of P2 receptors in TRT-HU1 cells.** Equal amounts of protein from each sample were loaded and HeLa cells were used as positive control. All P2 receptor polyclonal antibodies were purchased from Alomone Labs (Jerusalem, Israel) and used at a concentration of 1∶1,000. Anti-GAPDH monoclonal antibody was purchased from Fitzgerald Industries International (Acton, MA) and used at a concentration of 1∶25,000.
(TIF)
