# Supplementary figures and images for: Pannexin 1 Channels Play Essential Roles in Urothelial Mechanotransduction and Intercellular Signaling
Source: PLoS One. 2014 Aug 29;9(8):e106269. doi: 10.1371/journal.pone.0106269 (PMC4149561; doi:10.1371/journal.pone.0106269)

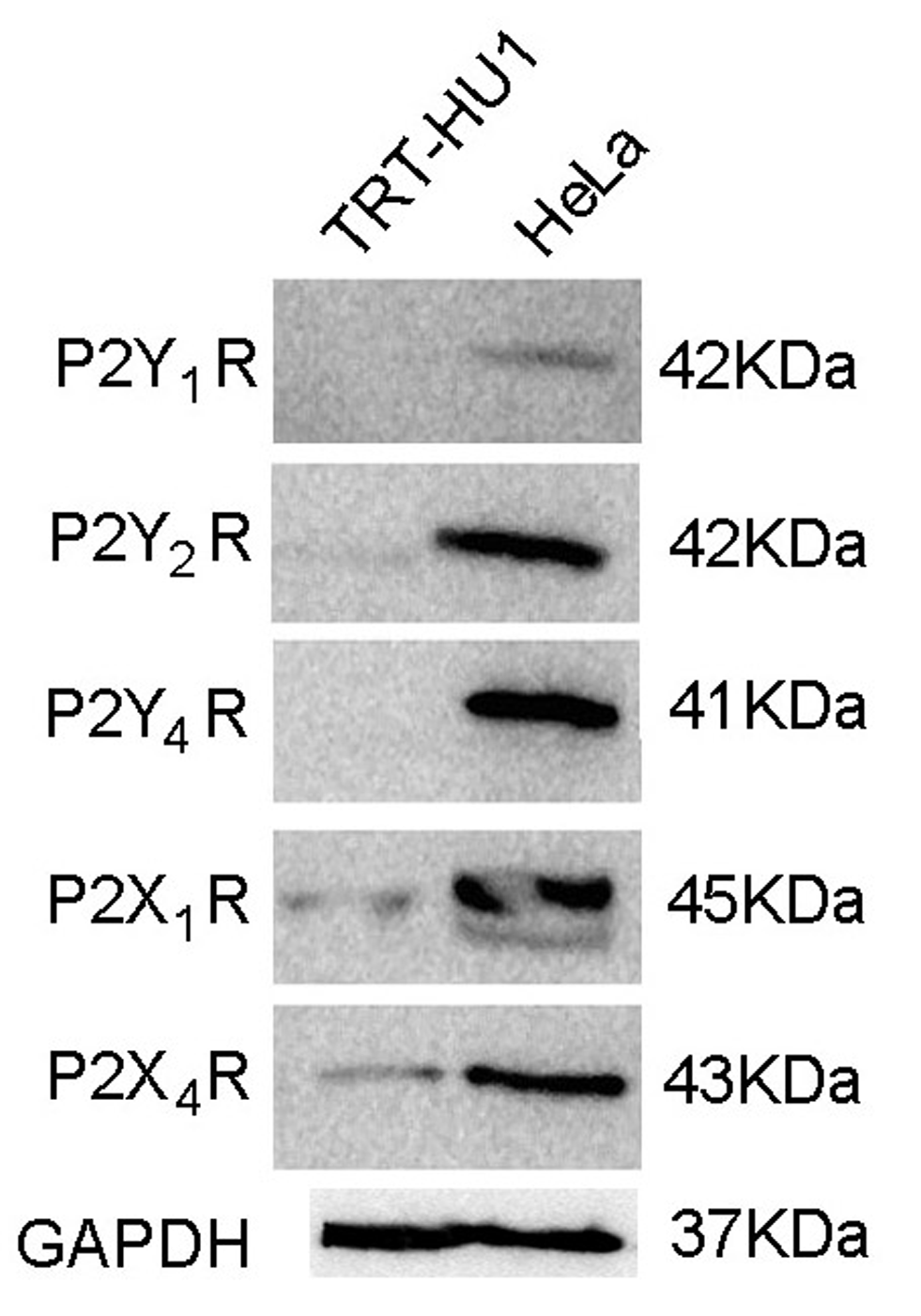

Supplement: Figure S1 — Representative immunoblots showing expression of P2 receptors in TRT-HU1 cells. Equal amounts of protein from each sample were loaded and HeLa cells were used as positive control. All P2 receptor polyclonal antibodies were purchased from Alomone Labs (Jerusalem, Israel) and used at a concentration of 1∶1,000. Anti-GAPDH monoclonal antibody was purchased from Fitzgerald Industries International (Acton, MA) and used at a concentration of 1∶25,000. (TIF) [file pone.0106269.s001.tif]
